# A proactive general practice: Integrating public health into primary care

**DOI:** 10.1080/17571472.2018.1445946

**Published:** 2018-03-20

**Authors:** Salman Rawaf

**Affiliations:** Department of Primary Care and Public Health, School of Public Health, Faculty of Medicine, Imperial College, London, UK

This year, we are celebrating 40 years since the WHO Alma Ata Declaration of *Health for All* first emerged [1]. In conjunction with the World Health Report 2008; *Primary Care: now more than ever*, the move from the disease model of primary care service to a more proactive model based on public health principles, is clearly needed [2]. Primary care, derived from General Practice, proved its value and effectiveness in reducing amenable mortality from communicable and non-communicable diseases, by reducing hospitalization and the use of emergency department visits, improving patient outcomes and helping to counteract the negative impact of poor economic conditions on health [3–5]. Indeed, countries oriented towards primary care have populations with better health and services that are delivered at lower costs [4,6,7]. Universal Health Coverage is one of the key targets for the UN 2015 Sustainable Development Goals (SDGs). However, such a target cannot be achieved without full primary care coverage for the entire population. We know that patients benefit, at all ages, from proactive approaches by general practitioners and their teams through listening, asking questions, providing information, and intervening to protect health and prevent disease as well as providing diagnosis, treatment, and continuous care. For us in public health, general practice is the front line to promote and protect health and prevent disease. The NHS general practice gratified itself as one of the first systems in the world that moved from a disease model to health model [8]. The immunization and vaccination programme; systematic screening in all ages; the Quality and Outcome Framework (QOF); The NHS Health Check; and talking therapies are examples of integration of public health into primary care. In the last decade, Brazil has managed to introduce a new model of integration by adopting the British General Practice model. Many public health functions are delivered through community health workers attached to primary care. These workers, with a workload of about 140 families each, are involved directly with families in supporting chronic disease management, triaging conditions like anaemia or dehydration, managing disease-specific programs such as for tuberculosis, providing sexual health advice, pre- and post-natal care, including breastfeeding assistance, child development assessment, cancer screening, supporting immunization programs, infectious disease monitoring, and health promotion advice [9]. This approach has proved to be very successful in reducing the burden of disease and improving health [10,11]. Other health systems in rich countries are following in this direction, however this is not the case for low and middle-income countries where most of health systems remain static.

In 2012, I proposed five possible models to integrate public health into primary care [12]. These include: Integrating public health professionals into primary care (representatives from both organizations working together in same setting) [13];Incorporating public health functions within primary care settings, (including wellbeing and preventive services as part and parcel of the comprehensive and proactive benefit packages) [8]; Incorporating primary care services within public health settings; Building public health incentives into primary care; and primary care staff that are trained in public health (dual training or a family physician with special interest in public health) [14,15]. Now as then, I believe that it is essential to train GPs in public health if we are to deliver the proactive comprehensive primary care needed to meet the health needs of the population in the 1^st^ Century. Such needs are becoming more complex and translate, on many occasions, into immediate demands on services. It is fuelled by population growth, the ageing population and rapid advancement in technology and medicine. In the last decade, the Royal College of General Practitioners has put huge professional and political pressure on practitioners and UK Government to change the emphasis towards a holistic approach to health [16]. They have stated that GPs play a crucial role in promoting health and preventing disease. Its curriculum statement on health promotion encourages GPs to be proactive in consultations to, for example, ‘discuss healthy living with the patients and for the early detection of illness’ and ‘provide appropriate diagnostic, therapeutic and preventative services to individuals, and to the registered population’ [16]. This is welcomed, but is not a replacement for formal training in another speciality: public health medicine.

This Issue of LJPC reveals practical ways to combine the skills of primary care and public health at local level. Banarsee et al discuss how clusters of general practices in localities of about 50,000 population can work in partnership with public health practitioners to support self-care, and to evaluate integrated working between primary care and mental health specialists for people with severe and enduring mental illness.

Such a public health/primary care partnership may also assist Roberts’ call for integrated working between respiratory specialists and community-based GPs, nurses and pharmacists; such integration also needs public health skills to evaluate ‘patient reported outcomes (PROMs) with generic tools such as SF-36, EQ-5D, COOP and HUI as well as COPD-specific instruments including CRQ, CCQ and SQR’.

Papanikitas and Lunan describe a need for interdisciplinary reflection on ethical aspects of practice, including guidelines. Public health needs to be included in such reflections as well, and this too should happen in geographic localities, to build a local, reflective community for health and care.

Oliviera, in his description of the Republic of Macedonia, shows another example of how the roles of healthcare practitioners and public health complement each other to describe the story of a vulnerable country and its health. All of these papers reveal the multi-layered advantage of combining public health and primary care skills at local level.

Some may argue that general practice is already overstretched due to the factors mentioned above, so collaborating with public health and others, however valuable, is too much to consider. An alternative argument says that the advantages of providing comprehensive services to all ages will be cost-effective overall and we need to pay attention to the linkages. Also let us acknowledge the inadequacy of the number GPs we have in the UK for frontline service, and also the poverty of their training in collaborative, integrating processes. The current formula of 6 GPs per 10,000 people is dated and service needs to be expanded. Technology, as some believed, did not reduce the demand on services. With increasing health literacy, people are having the foresightto promote and protect their health rather than to be ensued by illness. Public awareness and understanding of technology in medicine and health is leading to greater demand and proactivity in seeking service, whether by asking a clinician to provide a particular technology or by the regular use of technological devices for managing or tracking health, such as Apps and the Internet. It suggests that the huge advancements in technology within a relatively short timeframe could have large implications for delivery of health and social care services, and that further workforce would need to be trained to deliver more efficient services designed to meet the needs of both the population and the individual [17].

The NHS and the respective general practice is the envy the world, where many countries could learn from our experience. It is now recognised that a health system, which is not primary care-led is weak and expensive and primary care without fully trained family physicians is of poor quality. Countries that put greater emphasis on proactive primary care with public health skills are better able to help reduce the inequities in health care within the population and provide comprehensive care in today’s complex world.

## About the author

SR is a Professor of Public Health and served in the NHS as a Director of Public Health for 23 years, with extensive experience in education and training.

## Funding

This work was supported by WHO EURO for research on integration of public health into primary care.
